# A Series of Efficient Umbrella Modeling Strategies to Track Irradiation-Mutation Strains Improving Butyric Acid Production From the Pre-development Earlier Stage Point of View

**DOI:** 10.3389/fbioe.2021.609345

**Published:** 2021-06-16

**Authors:** Li Cao, Yue Gao, Xue-Zhen Wang, Guang-Yuan Shu, Ya-Nan Hu, Zong-Ping Xie, Wei Cui, Xiao-Peng Guo, Xiang Zhou

**Affiliations:** ^1^College of Life Sciences and Engineering, Hexi University, Zhangye, China; ^2^Institute of Modern Physics, Chinese Academy of Sciences, Lanzhou, China; ^3^College of Life Science, University of Chinese Academy of Sciences, Beijing, China

**Keywords:** *Clostridium tyrobutyricum*, butyric acid, MCMC model, logistic regression, luedeking—piret model, fermentation

## Abstract

*Clostridium tyrobutyricum* (*C. tyrobutyricum*) is a fermentation strain used to produce butyric acid. A promising new biofuel, n-butanol, can be produced by catalysis of butyrate, which can be obtained through microbial fermentation. Butyric acid has various uses in food additives and flavor agents, antiseptic substances, drug formulations, and fragrances. Its use as a food flavoring has been approved by the European Union, and it has therefore been listed on the EU Lists of Flavorings. As butyric acid fermentation is a cost-efficient process, butyric acid is an attractive feedstock for various biofuels and food commercialization products. ^12^C^6+^ irradiation has advantages over conventional mutation methods for fermentation production due to its dosage conformity and excellent biological availability. Nevertheless, the effects of these heavy-ion irradiations on the specific productiveness of *C. tyrobutyricum* are still uncertain. We developed non-structured mathematical models to represent the heavy-ion irradiation of *C. tyrobutyricum* in biofermentation reactors. The kinetic models reflect various fermentation features of the mutants, including the mutant strain growth model, butyric acid formation model, and medium consumption model. The models were constructed based on the Markov chain Monte Carlo model and logistic regression. Models were verified using experimental data in response to different initial glucose concentrations (0–180 g/L). The parameters of fixed proposals are applied in the various fermentation stages. Predictions of these models were in accordance well with the results of fermentation assays. The maximum butyric acid production was 56.3 g/L. Our study provides reliable information for increasing butyric acid production and for evaluating the feasibility of using mutant strains of *C. tyrobutyricum* at the pre-development phase.

## Introduction

Butyrate (C_4_H_8_O_2_) possesses a structure of saturated tetra-carboxylic acid; the name, butyric acid is derived from the Latin word for butter (Gutteridge, 1981; Ren et al., 1997; Bettelheim et al., 2012). Butyric acid and its acid salts are used in numerous commercial products, including food additives and flavors, antiseptics, cellulose-based plastic products, drug formulations, and aromatics (Zigová and Sturdik, 2000; Agler et al., 2011; Jiang et al., 2011). Further, butyric acid can be used to prepare various butyrate esters. Methyl butyrate, which is a low-molecular-weight ester of butyric acid; it has a more pleasant aroma or better taste compared to those associated with butyric acid. Therefore, methyl butyrate is used as a food and perfume additive. Animal feed is also supplied in combination with methyl butyrate to reduce colonization of pathogenic bacteria. The use of butyric acid as a food flavoring has been approved by the European Union, and it has therefore been listed on the EU Lists of Flavorings in the EU FLAVIS database (Flavis number: 08.005). Butyric acid is generally present at concentrations of 82 mg/kg in candy, 60–270 mg/kg in chewing gum, 32 mg/kg in baked foods, 18 mg/kg in margarine, and 6.5 mg/kg in cold drinks. It has also been used as a fishing bait additive due to its powerful odor. Many commercially available flavors that are used in carp bait use butyric as their ester base. Butyric acid was previously produced through the oxidation of butyraldehyde [CH_3_ (CH_2_)_2_ CHO] (Henstra et al., 2007; Dagaut et al., 2009; Jin et al., 2011). However, customers tend to choose food additives, flavors as well as fragrances containing natural ingredients (Ragauskas et al., 2006; Nigam and Singh, 2011; Zhou et al., 2013a,b).

Butyric acid was previously widely produced through the oxidation of butyraldehyde. However, limited fossil resources and increasing environmental concerns have resulted in a switch to microbial fermentation, a green and renewable technology. A promising new biofuel, n-butanol, can be produced by the catalysis of butyrate, which can be obtained through microbial fermentation (Munasinghe and Khanal, 2010). Previous studies have demonstrated that *Clostridium tyrobutyricum (C. tyrobutyricum)* produces more butyrate than other strains. *C. tyrobutyricum* produces a relatively large amount of butyrate from five- or six-carbon sugars with high efficiency. Recently, research has identified improved *C. tyrobutyricum* strains (compared to traditional mutation and molecular biology methods). Mutagenic technology is reliable and is widely used for strain improvement. Various mutation approaches are commonly employed in mutational engineering to produce bacteria with novel traits; these include chemical and physical mutagenesis. Afterward, bacteria strains produced using such technologies are screened by selective medium to identify mutant strains with remarkable performance and application value. Because of the frequent use of traditional mutagenic approaches, several engineering strains have build up tolerance. Hence, investigators have begun to establish novel methods for enabling microbe mutagenesis; heavy ion beam irradiation, considered as a highly efficient irradiating method, has been employed in a variety of studies aimed at developing novel bacterial strains using mutagenesis, and this strategy has achieved remarkable success with good economic benefits (Table 1). At present, fermentation-derived butyric acid plays an important role as a food additive. Microorganism fermentation engineering is the cornerstone of bioengineering and biotechnology. It is the fundamental application of biotechnology and its use is at the core of the biotechnology industry. Butyric acid fermentation processes have been limited by two factors, i.e., the production performance of *C. tyrobutyricum* with respect to butyric acid, and the fermentation conditions. In recent decades, the fermentation industry has developed rapidly, and its scale has grown. In order to obtain maximum efficiency from industrial-scale production, we must ensure optimal conditions for *C. tyrobutyricum* to grow and synthesize metabolites. Therefore, the optimization of fermentation conditions is becoming increasingly important. Previous studies have demonstrated that *C. tyrobutyricum* produces more butyrate than other strains (Lan and Liao, 2012; Jang et al., 2012, 2014). *C. tyrobutyricum* produces high levels butyrate from five- or six-carbon sugars (Tao et al., 2008; Peralta-Yahya et al., 2012; Zhou et al., 2014a) with high efficiency. Recently, *C. tyrobutyricum* strains with improved performance compared to that of *C. tyrobutyricum* strains produced using traditional mutation and molecular biology methods have been developed (Choi et al., 2012; Dwidar et al., 2012; Pappu et al., 2013). Artificial manipulation of traditional mutation strategies used for butyrate production was unable to result in the production of mutants with satisfactory butyric acid mass production abilities. Thus, it cannot be used as a suitable technique for scaling up industrial fermentation (Yu et al., 2011; Mattam and Yazdani, 2013; Zhou et al., 2017).

**TABLE 1:** Traditional mutagenesis methods and heavy-ion irradiation with the reported mutation methods are widely used in strain improvement.

Mutants	Sources	Mutant methods	References
*Aurantiochytrium* sp. T-99	*Aurantiochytrium* sp. CGMCC 6208	Heavy-ions mutagenesis	Cheng et al., 2016
*Trichoderma viride* CIT 626	*T. viride* GSICC 62010	^12^C^6+^-ion beam irradiation and Electron beam irradiation	Li et al., 2016
*Streptomyces fungicidicus* M30	*S. fungicidicus* SG-01	Heavy ion mutagenesis	Liu et al., 2018
*Chlorella* K05	*C. pyrenoidosa* FACHB-9	Heavy-ion irradiation mutagenesis	Song et al., 2018
*Mortierella alpina* F-23	*M. alpina* SD003 (CGMCC No.7960)	Heavy ion mutagenesis	Zhang et al., 2018
*Lactobacillus thermophilus* A69	*L. thermophilus*	Heavy ion irradiation	Tian et al., 2019
*Streptomyces avermitilis* 147-G_5_8	*S. avermitilis* AV-J-AO	Heavy-Ion beam Irradiation	Wang et al., 2017
*Arthrobacter strain* C2	*A. strain* C1	^12^C^6+^ heavy-ion beam	Li et al., 2018
*Blakeslea trispora* WY-239	*B. trispora* NRRL 2895	Mutation with ARTP	Wang et al., 2020
Chiba maru No. 10	*Colocasia esculenta* L. Schott	Heavy-ion beam irradiation	Matsuyama et al., 2020
Gamma ray-induced mutants (M_7_)	Rice (*Oryza sativa* L.)	Gamma Ray	Hwang et al., 2020
*Clostridium carboxidivorans P7-EMS*_*III*__–__J_	*C. carboxidivorans* (P7)	Ethyl methanesulfonate (EMS)	Lakhssassi et al., 2020
*Oryza sativa* L. *m3*	*Indica* landrace BBS.	Carbon ion irradiation	Peng et al., 2019
*Aspergillus fumigatus* MS160.53	*A. fumigatus* MS13.1	Heavy ion beam mutagenesis	Dong et al., 2019
*Aspergillus niger* H_11201_ *Clostridium tyrobutyricum* No. H51–8–4	*A. niger* H_11_ *C. tyrobutyricum* strains ATCC 25755	^12^C^6+^ ion beam	Jiang et al., 2016
*Yarrowia lipolytica* Mut-96	*Y. lipolytica* Wt-11	Low-energy ion implantation	Ping et al., 2018
*Monascus purpureus* KS301U and KS302U	*M. purpureus* KUPM5	UV (Ultraviolet) irradiation and NTG (N-methyl-N’-nitro-N-nitrosoguanidine) treatment	Ketkaeo et al., 2020
*C. tyrobutyricum* C.T^UV^	*C. tyrobutyricum* DSM 2637	UV irradiation, nitrous acid, and ethidium bromide treatment	Akhtar et al., 2018

One breeding tactics were put forward with respect to existing situation, as an important breeding method, heavy ion beam radiation mutagenesis breeding has the advantages of high mutation rate, wide mutation spectrum, stable mutants, and short breeding cycle. Induced heavy ion beam radiation mutagenesis is one of the most effective and economy means for the proper utilization of the existing well-characterized *C. tyrobutyricum* strains resources, especially self-owned traits improvement and enhancement without altering good optimization-genetic background of the strains. The heavy ion beam radiation mutagenesis has enormous potential to accelerate the mutation breeding in *C. tyrobutyricum* strains, it will be become fully aware of as a fact that rapid improvement of *C. tyrobutyricum* quality and its yield of butyric acid. Rheological properties of the bioprocessing and the downstream manufacturing of butyric acid are greatly rely on the production capacity of mutant *C. tyrobutyricum*, therefore cautiously designed ^12^C^6+^ heavy-ion radiation indices are required. An appropriate mathematical model is the basis and prerequisite for process control and optimization. Toward this, in the context of ^12^C^6+^ heavy-ion radiation the first step is the modeling of ^12^C^6+^ heavy-ion radiation parameters and the fermentation process, and the second step is the process control and optimization. In this study, the ^12^C^6+^ heavy-ion radiation has been used for bioprocess and biological fermentation devisal and scale-up cultivation. Associated empirical models aimed at the biological fermentation of mutant strains have been proposed to optimize operation conditions for mutational strain growth, butyric acid generation, and glucose consuming, because promoting butyric acid production is vital for future utilizations of the process in bio-fermentation field.

## Materials and Methods

### ^12^C^6+^ Heavy-Ion Beam Irradiation

Previous studies have been reported the heavy-ion radiation experimental setups (Zhou et al., 2014b). The extracting time of ^12^C^6+^ heavy ions (about 220 AMeV) was about 3 s, additionally the dosage of priming was 50–90 Gy. The dose rates were 5 Gy/min. Following operation parameters were used: energy input of radiation, 220 AMeV; the distance from ^12^C^6+^ heavy ion nozzle exits to spore suspensions, 4 mm; temperature of the ^12^C^6+^ heavy-ion beams, < 36–38°C. Inoculum was prepared as previous research reported by Roos et al. (1985) and Wiesenborn et al. (1988). Cell strains from an independent colony on a 3 × Reinforced Clostridial semi-defined P2 medium base plate (RCM-DRCM, AppliChem, Germany), were transferred to 250 mL of semi-defined P2 medium and cultured for 18 h.

### Strain Growth and Cultivation Medium

Wild-type *Clostridium. tyrobutyricum* (ATCC 25755) was obtained from the Biophysics Research Laboratory, Institute of Modern Physics, Chinese Academy of Sciences, China. The optimized culture medium included the following (g/L): yeast extract, 4.2; peptone, 3.1; K_2_HPO_4_, 2.7_;_ KH_2_PO_4_, 3.6; MgSO_4_, 0.3; MnSO_4_, 0.25; FeSO_4_, 0.03; NaCl, 0.03; yeast extract, 1.6 (Difco, Detroit, MI, United States); ammonium acetate, 2.5; *p-*aminobenzoate, 0.0003; thiamin, 0.0003; biotin, 5 × 10^–5^; thiamphenicol, 3.5 × 10^–5^. Samples were stored in an anaerobic chamber. The inoculum preparation and batch anaerobic fermentations were conducted in the 7-L BioFlo^®^/CelliGen^TM^ 115 fermentor/bioreactor (New Brunswick Scientific Co., Edison, NJ) including 270 mL inoculum added to 2 L of reinforced clostridial semi-defined P2 medium. The P2 medium contained the following ingredients (g/L): yeast extract powder, 1; KH_2_PO_4_, 0.5; K_2_HPO_4_, 0.5; para-aminobenzoic acid, 0.001; thiamin, 0.001; biotin, 1 × 10^–5^; MgSO_4_7H_2_O, 0.2; MnSO_4_7H_2_O, 0.01; Fe_2_SO_4_7H_2_O, 0.01; NaCl, 0.01; and ammonium acetate, 2.2. The initial glucose concentrations were 0–180 g/L. The temperature, pH value, and agitating speed remained at 36–38°C, 6.0–6.2, and 160 rpm, respectively. A nitrogen without oxygen supply rate of 60–65 mL/min was maintained. The optimal concentrations were 650 g/L of glucose as well as 22.5 g/L of MgSO_4_⋅7H_2_O.

### Analysis Methods

The system of high-performance liquid chromatography (HPLC) was applied to detect and analyze carbohydrates (containing glucose) in the liquid medium for fermentation. This HPLC system was comprised of an auto-injector (Agilent 1100, G1313A), a Zorbax carbohydrate analytical column (250 mm × 4.6 mm, 5 μm; Agilent, United States), a high-pressure pump (Agilent 1100, G1311A), a refractive index detector (Agilent 1100, G1362A) as well as a column oven retained at 30°C (Agilent 1100, G1316A). Ethyl nitrile was selected as mobile phase (the ratio of water/ethyl nitrile = 1:3), and the flow rate was 1.5 mL/min. Butyrate and acetate were detected by a gas chromatograph (GC) (Shimadzu, Columbia, MD, United States, GC-2014) which was furnished with a fused silica column (0.25 mm film thickness and 0.25 mm ID, 30 m, Stabilwax-DA) as well as a flame ionization detector. The injection temperature of GC was 200°C, and per microliter sample was injected through an automatic injector (Shimadzu, AOC-20i). The temperature of column remained at 80°C within 3 min, then increased to 150°C at a rate of 30°C per minute, eventually remained at 150°C lasting 3.7 min. Moreover, the cell density was analyzed via testing the optical density values (OD) of the cell suspensions at 600 nm by a UV-spectrophotometer (Genesys 20, Thermo Scientific, United States) with a transition of 0.412 ± 0.012 g/L of dry cell weight (DCW) for each OD unit. The levels of elemental carbon (C), hydrogen (H), oxygen (O) and nitrogen (N) were detected by Sercon-GSL (CE Instruments, Milan, Italy).

### Survival Fraction Determination: Mtt Assay

The survival fraction can be identified as previously noted (Buch et al., 2012). Hundred microliter MTT reagent (0.25 g/L) per well was added into Dulbecco’s revised Eagle’s medium (DMEM, Gibco Glasgow, United Kingdom), and cultured at 36–38°C for 40 min. MTT experiment was conducted by 128-well plates which contained 5,500–7,500 cells for each well. The survival fraction was calculated using the following equation (Price and McMillan, 1990):

(1) $$S\,u\,r\,v\,i\,v\,a\,l\,\,f\,r\,a\,c\,t\,i\,o\,n=2^{-n},n=\frac{T_{d\,e\,l\,a\,y}}{T_{d\,o\,u\,b\,l\,i\,n\,g\,t\,i\,m\,e}}$$

where *T*_*delay*_ is the time for achieving the specific absorption values of the control groups vs. the irradiation cells, while *T*_*doubling time*_ is the time needed for doubling the content of cells.

### Monod Kinetic Model

The Monod equation is regarded as a kinetic model which exhibits microbe growth as a function of specific growth rate and an essential substrate concentration (Lobry et al., 1992). The Monod model was modified by introducing a substrate inhibition term (Drapcho, 2006):

(2) dXdt=μX=μmS(S+Ks+S2KI)×X

(3) dXdt=μX=μmS(S+Ks+S2KI)×(1−PPd)iX

where *X* is the dry cell weight (g/L), μ represents the growth rate (1/h), μ*_*m*_* represents the maximal growth rate (1/h), *S* means the growth-restricting substrate concentration (g/L), *K*_*S*_, and *K*_*I*_ are constants of substrate saturation and substrate inhibition, respectively (g/L), *P* means the products concentration (g/L), *P*_*d*_ represents the product concentration without cell growth, then *i* represents the inhibitory extent of product. In this study, *P* is the total amount of butyric acid (g/L), and *P*_*d*_ is the total amount of acetic acid (g/L).

### Luedeking—Piret Model

The Luedeking—Piret model is a microbe growth model possessed a growth-related portion as well as a non-growth-related portion. The Luedeking–Piret model can be revised by introduction of a term of substrate inhibition (Luedeking and Piret, 1959; Kubicek et al., 1985):

(4) $$\frac{d\,P}{d\,t}=\alpha \,\frac{d\,X}{d\,t}+\beta \,X\,\left(1-\frac{\left[H\,L\right]}{H\,L_{i\,n\,h}}\right)$$

We rewrote the formula as follows:

(5) $$\frac{d\,P_{1}}{d\,t}=\alpha_{1}\,\frac{d\,X}{d\,t}+\beta_{1}\,X,\,\frac{d\,P_{2}}{d\,t}=\alpha_{2}\,\frac{d\,X}{d\,t}+\beta_{2}\,X$$

where *P*_1_ is the concentration of butyrate (g/L), *P*_2_ is the concentration of acetate (g/L), α is cell growth related (g/g), and β is non-growth-related (g/g/h).

### Statistical Analysis

Several mathematical formulas, such as the Jacobian matrix of the vector function, the sum-of-squares function, loglog counting, logistic regression as well as Probit regression, were used to analyze predicted simulation values.

## Results and Discussion

### ^12^C^6+^ Heavy-Ion Energy Input and Dose Dominating Survivors

Several experiments were conducted using varying irradiation dose parameters (50–80 Gy), and the survival rates (10.2–84.7%) were in comparison with a representative group of experimental results to determine the optimal ^12^C^6+^ heavy ions for 220-AMeV energy input and the appropriate dose. Based on the strain characteristics and survival rates, sample points were randomly assigned prior to any data-processing tasks. This selection strategy helped avoid bias from the human operator. The heavy-ion beam irradiation experiments were classified into three groups of seven samples each (Table 2), No. Q36–8–1, No. S24–3–2, No. H51–8–3, H51–8–4, No. S24–3–5, No. Q36–8–6, and Q36–8–7. These experiments were composed of 21 independent irradiating tests and their results. The survival probability decreased upon increasing the irradiation dose (Table 2). A total of *n* = 5,900 cell strains were irradiated, eventually causing 900 of cell death. In strains subjected to random ^12^C^6+^ heavy-ion irradiation, *n* = 5,900 strains received the lowest irradiation dose (50 Gy), and the survival proportion varied from 0 to 1. We modeled cell strain lethality as a function of irradiation dosage by means of a regression model and a −2 log-likelihood function. If *y* is the number of dead cells (strains), then *y* can be used as a random variable along with a binomial distribution expressed by the formula as shown below:

**TABLE 2:** Random sampling locations after ^12^C^6+^ heavy-ion irradiation with an energy input of 220 AMeV and a dose of 50∼80 Gy.

Sample No. Q36-8	Irradiation dose	Log of irradiation dose	Total of cells strains	Total of cells strains lethal	Survival proportion
1	50 Gy	1.6989	5,900	900	0.8475
2	55 Gy	1.7403	6,250	2,470	0.6048
3	60 Gy	1.7782	5,500	2,940	0.4655
4	65 Gy	1.8129	5,600	3,250	0.4196
5	70 Gy	1.8451	6,300	5,170	0.1794
6	75 Gy	1.8575	5,900	5,300	0.1017
7	80 Gy	1.9031	6,200	6,200	0

**Sample No. S24–3**	**Irradiation dose**	**Log of irradiation dose**	**Total of cells strains**	**Total of cells strains lethal**	**Survival proportion**

1	50 Gy	1.6989	6,100	740	0.8787
2	55 Gy	1.7403	6,000	2,300	0.6167
3	60 Gy	1.7782	5,900	3,150	0.4661
4	65 Gy	1.8129	5,600	3,270	0.4161
5	70 Gy	1.8451	6,300	5,200	0.1746
6	75 Gy	1.8575	6,200	5,490	0.1145
7	80 Gy	1.9031	6,100	6,100	0

**Sample No. H51–8**	**Irradiation dose**	**Log of irradiation dose**	**Total of cells strains**	**Total of cells strains lethal**	**Survival proportion**

1	50 Gy	1.6989	6,200	730	0.8823
2	55 Gy	1.7403	5,500	3,430	0.6236
3	60 Gy	1.7782	6,200	3,200	0.4839
4	65 Gy	1.8129	5,600	3,100	0.4464
5	70 Gy	1.8451	5,500	4,560	0.1709
6	75 Gy	1.8575	6,300	5,600	0.1111
7	80 Gy	1.9031	5,600	5,600	0

(6) $$P\left(y=k\right)=\left(\begin{array}{c} n\\ k \end{array}\right)p^{k}{\left(1-p\right)}^{n-k}$$

where *y* assumes a value called *k*, and *k* = 0, 1,…5,900. We thus have a binomial distribution where *p* (the success probability) depends upon the irradiating dose *x*:

(7) $$\text{p}\,\left(x\right)=\frac{e^{\beta_{0}+\beta_{1}\,x}}{1+e^{\beta_{0}+\beta_{1}\,x}}$$

where β*_0_* and β*_1_* = the regression parameters, and *p (x)* varies from 0 to 1. Then a new logistic regression function can be deformed from the formula (7) to:

(8) $$L\,o\,g\,i\,t\,\left(p\,\left(x\right)\right)=L\,o\,g\,\left[p\,\left(x\right)/\left(1-p\,\left(x\right)\right)\right]=\beta_{0}+\beta_{1}\,x$$

This equation is the link function for the logistic regression. In the random ^12^C^6+^ heavy ion irradiated samples, the minimum irradiating dosage set as *x*_1_ = 50 Gy; *n_1_* = 5,900 cell strains were irradiated in the dose above, leading to *y_1_* = 900 cell strain deaths. The likelihood function is written as:

(9) $$L=\left(\begin{array}{l} {\text{n}}_{1}\\ y_{1} \end{array}\right)p{\left(x_{1}\right)}^{y_{1}}{\left(1-p\left(x_{1}\right)\right)}^{n_{1}-y_{1}}\times \cdots \times \left(\begin{array}{l} n_{m}\\ y_{m} \end{array}\right)p{\left(x_{m}\right)}^{y_{m}}{\left(1-p\left(x_{m}\right)\right)}^{n_{m}-y_{m}}$$

where *L* = as and β_0_ and β_1_, like in function (7). The parameters of logistic regression are following:

(10) $$\text{p}\,\left(x\right)=\frac{e^{\beta_{0}+\beta_{1}\,x_{\text{i}}}}{1+e^{\beta_{0}+\beta_{1}\,x_{i}}},f\,o\,r\,i=1,2,\ldots ,m.$$

**FIGURE 1:**
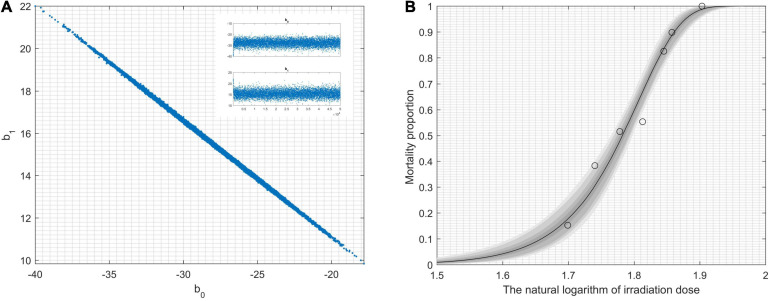
Results obtained by modeling lethality-associated a function of irradiation-dose, and applied delayed-rejection adaptive metropolis Markov chain Monte Carlo chain (MCMC). **(A)** 2D MCMC-chain using 5.0 × 10^4^ consecutive steps of delayed-rejection adaptive metropolis MCMC. Top right figures: the 1D parameter chains. **(B)** The model fit randomly selected the chain subsets, and the model-predictive envelope was developed. The gray protection system of the plot corresponds to 50, 90, 95, and 99% posterior regions.

During the statistical analysis, the coding work for the Markov chain Monte Carlo model and the delayed rejection and adaptive metropolis portion were used as previously described (Haario et al., 2006; Dobson and Barnett, 2008). We aimed to model lethal situations in which the proposed distributions are selected with excessively large or small variances related to the target distribution. We performed the runs repeatedly with increasing dimensions. In order to obtaining credible random experimental results, two-dimension Markov chain Monte Carlo chain as well as one-dimension parameter chain lengths can be set at 50,000 steps for whole dimensions (Figure 1A). For Figure 1A, enlarged the “Top right figures: the 1D parameter chains” (Supplementary Figure 1). Each dimension was required to repeat 500 times. The simulations began by randomly generating a point from the experimental data. Simple linear regression parameters were found, including the intercept and slope from Equations (7) and (8). The maximum value of the log-likelihood function (*D* = 2[*l*(*b*_*max*_)−*l*(*b*)]) for the maximal model was -40.28. The data helped create a logistic regression model that was used to identify a correlation between radiation-related mortality of strain cells (*C. tyrobutyricum*) and ^12^C^6+^ heavy ions with 220 AMeV energy input as well as a dosage of log (50–80 Gy); meanwhile the model also resulted in the production of an approximated logistic regression curve (Figure 1B). The gray regions correspond, respectively, to 50, 90, 95, and 99% of posterior regions. The predictions of the model were consistent with the results of the *n*-independent irradiation tests (and radiation results). The probability *(p)* of success was identical for each irradiation test. Random ^12^C^6+^ heavy-ion radiation is a time-consuming technique and is not conducive to use in industrial applications. Thus, a carefully designed ^12^C^6+^ heavy-ion irradiation model is invaluable and can be widely used for *C. tyrobutyricum* irradiation in industrial applications.

### Determination of Positive or Negative Mutants

The generation of improved *C. tyrobutyricum* strains using mutagenesis and selection represents a better technique compared to traditional mutation methods. Molecular biology methods are essential to commercial exploitation of microbe fermentation processes. Applied heavy-ion irradiation technology represents a new, green method to produce butyric acid. The performance of *C. tyrobutyricum* mutants must be determined for use in industrial applications. Previously, we had reported that high producing mutant strains exhibited survival rates of 10.2–11.7% after ^12^C^6+^ heavy-ion irradiation, and validated the expression of proteins (∼85 kDa molecular weight) in these mutants. A protein of approximately 90–106 kDa correlated with higher survival rates (10.2–11.7%) than those of the wild-type strain (Zhou et al., 2014a). The results revealed large variations in molecular weight. All mutants were identified as “*Positive”* or “*Negative”* mutants. Random ^12^C^6+^ heavy-ion irradiation cannot result in the mutation of the *ack* and *pta* genes or cause accurate and reproducible damage to individual organisms. A “*Positive”* mutant demonstrated an increased ratio of *Y*_*butyric acid*_:*Y*_acetic acid_. We selected mutants at random from the 75 Gy irradiated samples (No. Q36–8–6, No. S24–3–6, and No. H51–8–6), and mixed them. These blends of samples were named as No. QSH-M-F_80_ and divided into eight groups, additionally each group contained seven samples. From these, we randomly selected two samples (Table 3). Table 3 presents the *Y_butyric acid_/Y_acetic acid_* ratios (δ) for all mutants and for the wild-type *C. tyrobutyricum*. In the wild-type strain, δ was typically 3.05:1, 3.25:1, or 3.32:1, whereas in the mutants, δ exceeded 5.45:1. The butyric acid productivity of the *C. tyrobutyricum* mutants had *P* < 0.05 random error, based on the mean values of five-time measurement. Strains with an increased ratio exceeding 3.05:1 exhibited a δ similar to that of the wild-type strain and were identified as “*Positive”* mutants. The *R*_*M/T*_ was estimated at 19.8% and the *R*_*P/T*_ at 5.8% according to the detected specific productivities of 16 mutants and the colony-forming number of each group (Table 3). The “*Positive”* mutant QSH-M-F_7__5__–_6–1 demonstrated the highest-butyric acid production among the randomly sampled and detected strains. The productivity of this mutant was more than 1.72-fold greater compared to wild-type strains. The models used for the optimization of the “*Positive”* mutant QSH-M-F_7__5__–_6–1 growth and acid production will be detailed below.

**TABLE 3:** Comparison butyric and acetic acid productivity after mutation via ^12^C^6+^ heavy-ion irradiation with an energy input of 220 AMeV and a dose of 75 Gy at 37°C, pH = 5.5 and 6.0 during the first 70 h of fermentation (*n* = 5).

Group	Colony number	No. Sample	*R _*butyric acids*_* *	*R*_acetic acids_ *	δ**
		W	34.32 ± 0.13	11.24 ± 0.09	3.05:1
QSH-M-F_7__5_–1	9	QSH-M-F_7__5_–1–1	43.56 ± 0.19	10.37 ± 0.23	3.43:1
		QSH-M-F_7__5_–1–2	47.86 ± 0.12	9.78 ± 0.17	4.98:1
QSH-M-F_7__5_–2	15	QSH-M-F_7__5_–2–1	44.46 ± 0.15	10.17 ± 0.21	4.37:1
		QSH-M-F_7__5_–2–2	39.78 ± 0.23	9.64 ± 0.12	4.13:1
*P*	*24*				
QSH-M-F_7__5_–3	19	QSH-M-F_7__5_–3–1	40.78 ± 0.11	12.56 ± 0.17	3.25:1
		QSH-M-F_7__5_–3–2	36.35 ± 0.21	12.78 ± 0.06	2.85:1
QSH-M-F_7__5_–4	17	QSH-M-F_7__5_–4–1	31.67 ± 0.23	13.42 ± 0.09	2.36:1
		QSH-M-F_7__5_–4–2	35.36 ± 0.32	11.87 ± 0.21	2.98:1
QSH-M-F_7__5_–5	8	QSH-M-F_7__5_–5–1	51.18 ± 0.16	10.27 ± 0.11	4.98:1
		QSH-M-F_7__5_–5–2	49.65 ± 0.23	9.62 ± 0.12	5.16:1
QSH-M-F_7__5_–6	23	QSH-M-F_7__5_–6–1	58.93 ± 0.27	10.78 ± 0.13	5.45:1
		QSH-M-F_7__5_–6–2	49.36 ± 0.21	9.92 ± 0.19	4.98:1
QSH-M-F_7__5_–7	9	QSH-M-F_7__5_–7–1	33.75 ± 0.13	12.35 ± 0.27	2.73:1
		QSH-M-F_7__5_–7–2	35.63 ± 0.09	11.21 ± 0.17	3.18:1
*M*	81				
QSH-M-F_7__5_–8	328	QSH-M-F_7__5_–8–1	53.36 ± 0.18	11.36 ± 0.09	4.70:1
		QSH-M-F_7__5_–8–2	35.67 ± 0.24	12.31 ± 0.18	2.90:1
*T*	*409*				
*R_*M/T*_* = *M/T* = *19.8%*			*R_*P/T*_* = *P/T* = *5.8%*		

* R_*butyric acid*_ and *R_*acetic acid*_ represent the percentages of the specific productivity of all mutants. R_*butyric acid*_ and R_*acetic acid*_ represent the percentages of the wild-type strain C. tyrobutyricum (W). **δ indicates the Y_*butyric acid*_:Y_*acetic acid*_ ratio. P represents the total colony number of positive mutants. M represents the total number of mutants, including positive and negative mutations. T represents the total colony number of P and M.

### “*Positive*” Mutant Growth

Biofermentation was investigated using varying glucose concentrations (5, 10, 15, 20, 25, 50, 75, 100, 125, 150, and 175 g/L). To identify he kinetic parameters of the growth models of the “*Positive”* mutants, the sum-of-squares function between experimental results gained from batch fermentations and predicted results of the Monod model need to be minimized. The Monod model is expressed by the following system of non-linear equations:

(11) $${\text{y}}_{i}=\frac{\theta_{1}\,x_{i}}{\theta_{2}+x_{i}}+e_{i}$$

where *y*_*i*_ represents the growth rate (1/h) obtained at substrate concentration *x*, θ*_1_* is the highest growth rate (1/h), and θ*_2_* means the saturation constant (in units of the substrate concentration). The non-linear models estimate θ*_1_* and θ*_2_* at low initial glucose concentrations (5, 10, 15, 20, and 25 g/L) by simplifying Equations (1–5). The parameters related to the substrate, θ*_1_*_=_ *K*_*S*_ (Equation 1) *and*θ*_2_* = *K*_*I*_ (Equation 1), were identified from the data obtained during the early exp-date of the “*Positive”* mutants’ growth phase, when no excrete organic acid inhibition occurred. θ*_1_* and θ*_2_* were estimated by minimizing the sum-of-squares function:

(12) $$M\,i\,n\,i\,m\,i\,z\,e\,S=\sum {\left(y_{i}-\frac{\theta_{1}\,x}{\theta_{2}+x}\right)}^{2}$$

**FIGURE 2:**
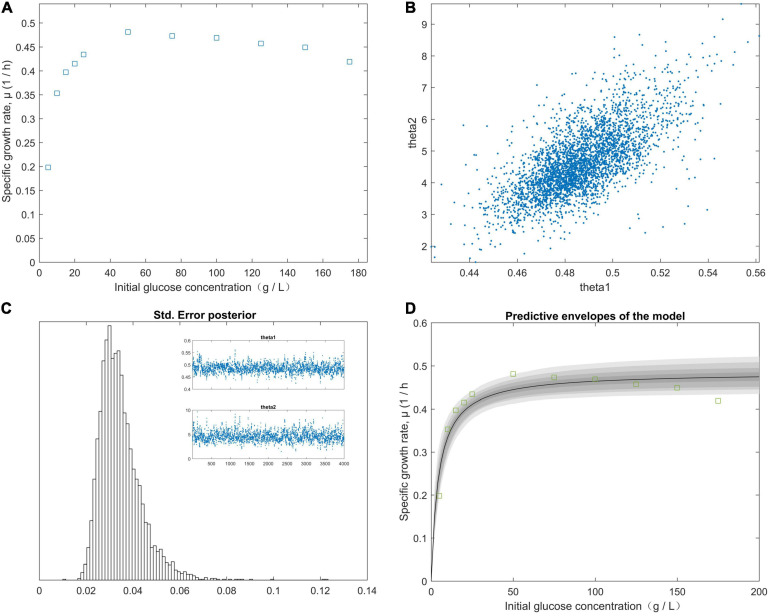
The effect of the initial glucose concentration gradient from predictive posterior distribution on the specific growth rate of the *C. tyrobutyricum* mutant. **(A)** The different specific growth rates of *C. tyrobutyricum* mutant correlated with the initial glucose concentration gradient. **(B)** Chain plots, plots 1 and 2, and dimensional marginal kernel density estimates of the posterior distributions. **(C)** Standard deviation was displayed at the square root of the s2chain. Top right figures: the 1D parameter chains with 4.0 × 10^3^ consecutive steps of delayed-rejection adaptive metropolis MCMC. **(D)** Predictive envelopes of the model: The gray protection system corresponds to 50, 90, 95, and 99% in the posterior regions as illustrated in the plot.

The right-hand panel (Figure 2D) shows the model fitted to the experimental data corresponding to the approximately 99% joint confidence region (Figure 2). Figure 2D was mapped using the sum-of-squares values calculated over a grid of paired experimental values for θ*_1_* and θ*_2_*. The initial values of the experimental parameters were estimated using the plotted data (Figure 2A). We minimized the sum-of-squares function using *fminsearch*. For our study data, *S_*R*_* = 0.0003, *n* = 5, *and p* = 2. The chain variable is the *nsimu* × *npar* matrix (Figure 2C). Chain plots, plots one and two, and the dimensional marginal kernel density approximations for posterior distributions were created. Plots 1 and 2 consisted of pairwise scatterplots of the columns of the chain (Figure 2B). The square root of the *s2chain* was obtained and used to determine the histogram of the chain error (standard deviation) (Figure 2C). A point estimate for model parameters can be computed from the average of the chain. We plotted the fitted model using the posterior means of the parameters (Figure 2D) and fit the fermentation data to the model predictions for high initial glucose concentrations (50, 75, 100, 125, 150, and 175 g/L). The values of the “*Positive”* mutants for the kinetic parameters μ*_*m*_*, *K*_*S*_, and *K*_*I*_ are listed in Table 4. As shown in Figure 2, the value of θ*_2_* (*K*_*S*_) was supposed to be less than 45 g/L, because θ*_1_* (μ*_*m*_*) was calculated from the plot of 1/*y*_*i*_ (1/μ) vs. 1/*x* (1/*S*). The initial glucose solution gradient was used to establish the predictive envelopes of the model. The variable *y*_*i*_ (μ) was fitted to the experimental data during the early exp-date growth phase of the “*Positive”* mutant, and revealed the original glucose solution gradient was higher than the glucose solution gradient in the culture medium. Because *y_*i*_* = θ*_1_* and $\text{S}=\sqrt{\theta_{2}}$from Equations (11) and (12), $S=\sqrt{K_{S}\cdot K_{I}}$ from Equation (1). The highest specific growth rate (θ*_1_*) of the “*Positive”* mutant would be obtained when the glucose gradient was approximate 50 g/L in the cultivation medium. A high dissolved CO_2_ concentration is a critical factor for achieving a high growth rate at a high cell density. The pathways of glucose metabolism in *Clostridium. tyrobutyricum* indicate that CO_2_ is released through the decarboxylic reaction of pyruvic acid to acetyl-CoA, leading to butyric and acetic acid production (Zverlov et al., 2006; Ezeji et al., 2007). The carbon mass balance based on the stoichiometric reactions is expressed as:

**TABLE 4:** Notations, units for Equations (2–5 and 15), parameters, data and constants.

Symbol	Description and units data and constants
*I*	Suppression degree of metabolite product 5.32
*K*_*d*_	Specific the strain of cell mortality rate (1/h) 0.0027
*K*_*I*_	Normal factor of substrate inhibition (g/L) 383
*K*_*S*_	Normal factor of substrate saturation (g/L) 1.71
*m*_*S*_	Maintenance coefficient of system (1/h) 0.015
*P*	Gradient of product of metabolism (g/L) –
*P*_*d*_	Critical gradient of product of metabolism (g/L) 53.8
*S*	Gradient of substrate (g/L) –
*X*	Gradient of biomass (g/L) –
*Y*_*Aa*_	Stoichiometric yield-factor of acetic acid (g/g) 0.997
*Y*_*Ba*_	Stoichiometric yield-factor of butyric acid (g/g) 0.973
*Y*_*X*_	Stoichiometric yield-factor of biomass (g/g) 0.812
***Greek letters***	
α*_*Aa*_*	Formation parameters of acetic acid associated with growth (g/g DCW) 0.83
α*_*Ba*_*	Formation parameters of butyric acid associated with growth (g/g DCW) 3.12
β*_*Aa*_*	Formation parameters of acetic acid associated with non-growth (g/g DCW/h) –
β*_*Ba*_*	Formation parameters of butyric acid associated with non-growth (g/g DCW/h) 0.049
*M*	Specific the strain of cell growth-rate (1/h) -
μ*_*m*_*	Maximum specific the strain of cell growth-rate (1/h) 0.48

(13) C6⁢H12⁢O6→C⁢H73⁢C⁢O⁢O⁢H+2⁢H2+2⁢C⁢O2

(14) $$C_{6}\,H_{12}\,O_{6}+2\,H_{2}\,O\to 2\,C\,H_{3}\,C\,O\,O\,H+4\,H_{2}+2\,C\,O_{2}$$

(15) $$-\frac{\text{d}\,S}{d\,t}=\frac{1}{Y_{X}}\,\frac{d\,X}{d\,t}+\frac{1}{Y_{B\,a}}\,\frac{d\,P_{B\,a}}{d\,t}+\frac{1}{Y_{A\,a}}\,\frac{d\,P_{A\,a}}{d\,t}+m_{S}\,X$$

These parameters are listed in Table 4. Gassing with N_2_ decreased the dissolved CO_2_ and H_2_, both of which evolved from the “*Positive*” mutant. The dissolution of CO_2_ in the culture medium is necessary for the biosynthesis of the components of “*Positive”* mutants. Increasing the growth rate of the mutant also increases the biomass concentration and productivity because the reaction of acetyl coenzyme A, and carbon dioxide is catalyzed via the specific enzyme acetyl-coenzyme A carboxylase. Substrate inhibition must be minimized before the growth of the mutant becomes retarded by the accumulated products in the bioreactor or scale-up. Butyric acid and acetic acid were the major and minor fermentation products, respectively. These acidic products inhibit growth and result in cell death (Martinez et al., 1998; Hanahan and Weinberg, 2011). Cell growth was previously demonstrated to be determined by *pK*_*a*_ (the dissociation constants for organic acids) and pH values of the cultivation medium (Cherrington et al., 1991; Narendranath et al., 2001). Butyrate and acetate have similar values of *pK*_*a*_ (4.93 and 4.83, respectively), additionally the pH value of the fermentation during the experiment remained at pH 6.0. The pH of the bioreactor was 6.0. The degrees of inhibition of the organic acids were identical. The degree of the product inhibition parameter (*i* = 5.32) was approximated from Equations (4) and (5) (Table 4). The growth of the “*Positive”* mutant decreased along with the bioreaction proceeded and stopped when the total quantity of acetic and butyric acids (*P*_*d*_) was 53.8 g/L. A drastic decrease in the biomass occurred at *K_*d*_* = 0.0027.

### Organic Acid-Specific Productivity of the “Positive” Mutant

**FIGURE 3:**
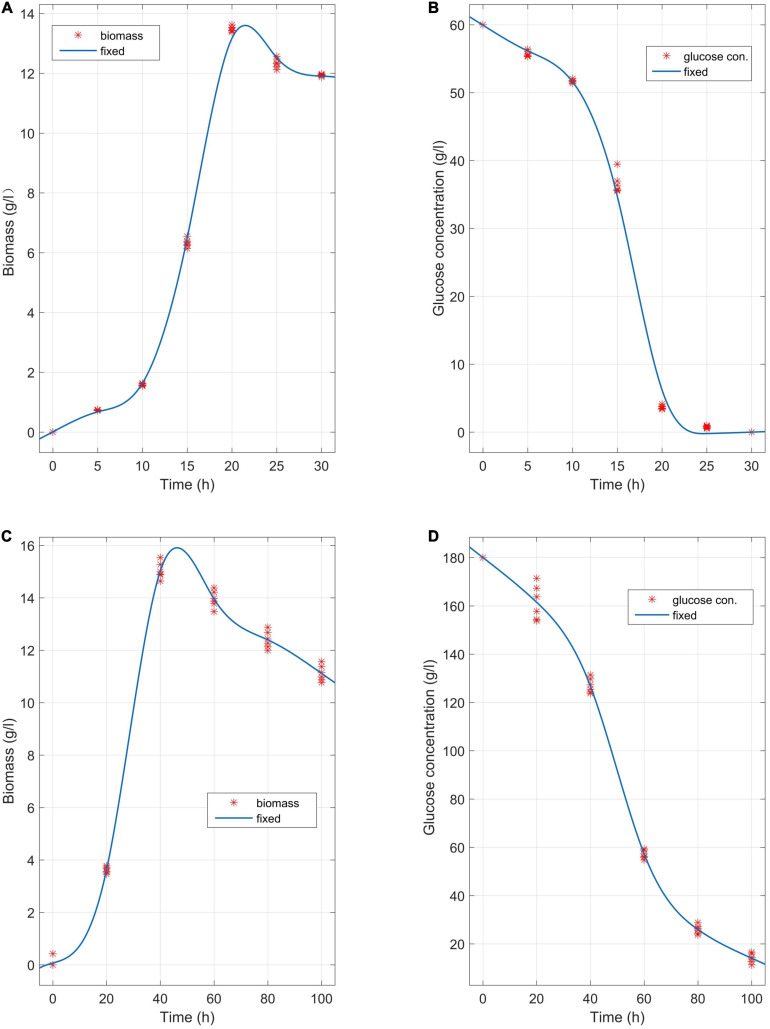
Biomass change and glucose consumption at different time points in the biofermentations of *C. tyrobutyricum* mutants in media with a concentration gradient of 60 g/L and 180 g/L glucose, respectively. **(A)** The mutants’ butyric and acetic acid productivity at 37°C, pH = 6.0 over 45 h of biofermentation (*n* = 6). **(B)** Glucose consumption (60 g/L) at different time points in the biofermentation of the *C. tyrobutyricum* mutant (45 h). **(C)** The mutants’ butyric and acetic acid productivity at 37°C, pH = 6.0 over 120 h of biofermentation (*n* = 6). **(D)** Glucose consumption (180 g/L) at different time points in the biofermentation of the *C. tyrobutyricum* mutant (120 h). Experimental (red asterisks); simulated (blue lines).

**FIGURE 4:**
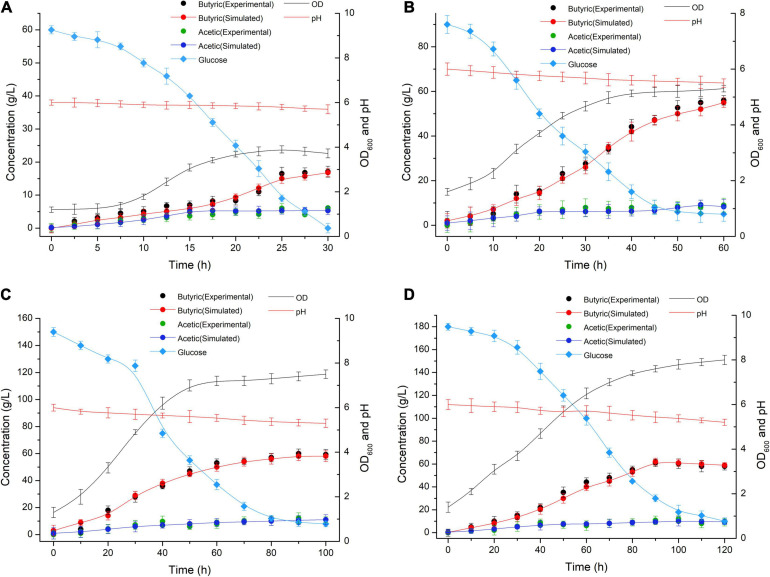
Butyric (Experimental: black symbols; Simulated: red symbols and red lines) and acetic (Experimental: green symbols; Simulated: blue symbols and blue lines) acid production at different time points in the biofermentations of the *C. tyrobutyricum* mutant in media containing glucose concentration gradients of **(A)** 60 g/L, **(B)** 90 g/L, **(C)** 150 g/L, and **(D)** 180 g/L glucose.

The media involving glucose concentrations of 60 g/L and 180 g/L were observed at different time points, and the biomass change and glucose consumption were measured during the biofermentation of *C. tyrobutyricum* mutants (Figure 3). α*_*Ba*_* = 0.83 (Equations 2–5 and 15) and α*_*Ba*_*_=_3.12 were estimated by fitting the biofermentation results of the model. The butyric acid-specific productivity of the “*Positive*” mutant was more affected by the μ parameter than the biomass change, as suggested by the higher value of the parameter β*_*Ba*_* compared to β*_*Aa*_*. Previous study has been suggested that butyrate can suppress cell growth. As organic acid concentrations reach a key value in the biofermentation process, cell death occurs (Muller-Feuga et al., 2004). Those “*Positive”* mutant cells dividing actively, mutant cells that exhibit a physiological lag stage, cells which are impaired and require repair prior to resolving lag and then those cells are dead. The lag phase durations for mutant cells in various physiological conditions metabolize different gradients of organic acids; exponentially growing wild-type cells have minimum stationary phases, lag phases, and longer lag stages in mutant strains. However, no gradual inhibiting effect of “*Positive”* mutant growth was noted. Indeed, no growth could be found when the biomass concentration exceeded 16 g/L or the butyric acid concentration exceeded 68 g/L (Figures 3, 4). The maximum biomass concentrations occurred at 20 and 40 h (Figures 3A,C). This result was slightly different from the results of model prediction. No significant differences were found in terms of glucose consumption between the two experimental conditions (Figures 3B,D). Additional energy expenditure can be used for the glucose transport through cellular membranes, which is powered by a phosphoenolpyruvate-controlling phosphotransferase system usually aimed at glucose transport. It is worth noting that more acetate can be generated from glucose, presumably to satisfy demand for higher ATP levels in glucose fermentation which could grow more quickly and greatly (Figure 4). A higher level of ATP can be produced when pyruvate generated from substrates is transformed into acetate instead of butyrate. These results indicated that the acetate/butyrate ratio improved depending on the growth rate, thus a low cell growth velocity was beneficial to the production of butyric acids. By calculating the stoichiometric Equations (13) and (14), the butyric acid yield gained from this research was approximate to the theoretical highest acetate/butyrate yield (0.489 g/g). These findings demonstrated that the metabolic burden was reduced for the “*Positive”* mutant. Our experimental results showed that the acetate/butyrate yield ranged from 0.27∼0.31 g/g for original glucose concentrations from 60 to 180 g/L. This observation is consistent with fast cell mutant growth and organic acid formation (Dawes and Ribbons, 1964; Barsanti et al., 2001). The obtained glucose consumption prediction model curves are consistent with the experimental data. The results of the maximum butyric and acetic acid productivities of the “*Positive”* mutant with different glucose concentration gradients (60, 90, 150, and 180 g/L) are shown in Figure 4. Butyric acid production ranged from15.5 to 56.3 g/L and acetic acid production from 4.8 to 13.5 g/L for original glucose concentrations from 60 to 180 g/L. Fine-grained differences were noted in the organic acid production between the assay data and the model predicted estimates (Figure 4). These differences may be attributed to the *m_*S*_* = 0.015 1/h (maintenance parameter) in Equation (15). This parameter was approximated through fitting the assay results to the above model. The *m*_*S*_ parameter during the lag phase was also assumed to be proportional to the biomass. Therefore, the established model cannot predict the lag phase accurately, which probably require further adjustment for parameters to better depict this phase.

## Conclusion

Factors influencing the irradiation-mutation strains that improve butyric acid production have been discussed based on the experimental results presented in this paper, and major modeling approaches concerning the estimation of butyric acid production have been critically estimated. In predictive irradiation-mutation strains, a multi-step modeling approach was employed. Primary models described the evolution of irradiation-mutation strain amounts along with duration, and they can be further divided into two groups: deterministic model and stochastic model. Primary deterministic models described the metabolism of mutation strains via a serie of deterministic model parameters. Among stochastic models, the parameters were identified as distributed variable or random variable. Secondary models were used to depict the relationship between the primary model parameters and affecting factors. In this study, a series of efficient umbrella modeling strategies were used to track and generate reliable data supporting the improvement of butyric acid production were presented. These modeling strategies confirmed the feasibility of using a mutant strain of *C. tyrobutyricum* at the pre-development phase.

## Data Availability Statement

The original contributions presented in the study are included in the article/Supplementary Material, further inquiries can be directed to the corresponding author/s.

## Author Contributions

LC and YG conceived, designed, and supervised the study. LC, YG, X-ZW, G-YS, Y-NH, Z-PX, WC, and X-PG performed the experiments, analyzed the data and contributed reagents, materials, and analysis tools. LC wrote the manuscript. LC and YG critically revised the manuscript. XZ final approval of the version to be published. All authors contributed to the discussion and comments on the manuscript and approved the submitted version.

## Conflict of Interest

The authors declare that the research was conducted in the absence of any commercial or financial relationships that could be construed as a potential conflict of interest.

## Supplementary Material

Supplementary Figure 1 For Figure 1A, enlarged the “Top right figures: the 1D parameter chains.”
