# Supplementary material for: A Series of Efficient Umbrella Modeling Strategies to Track Irradiation-Mutation Strains Improving Butyric Acid Production From the Pre-development Earlier Stage Point of View
Source: Front Bioeng Biotechnol. 2021 Jun 16;9:609345. doi: 10.3389/fbioe.2021.609345 (PMC8242359; doi:10.3389/fbioe.2021.609345)
Supplement: Supplementary Figure 1 — For Figure 1A, enlarged the “Top right figures: the 1D parameter chains.” [file Data_Sheet_1.PDF]

# *Supplementary Material*

## 1 Supplementary Figures and Tables

### 1.1 Supplementary Figures

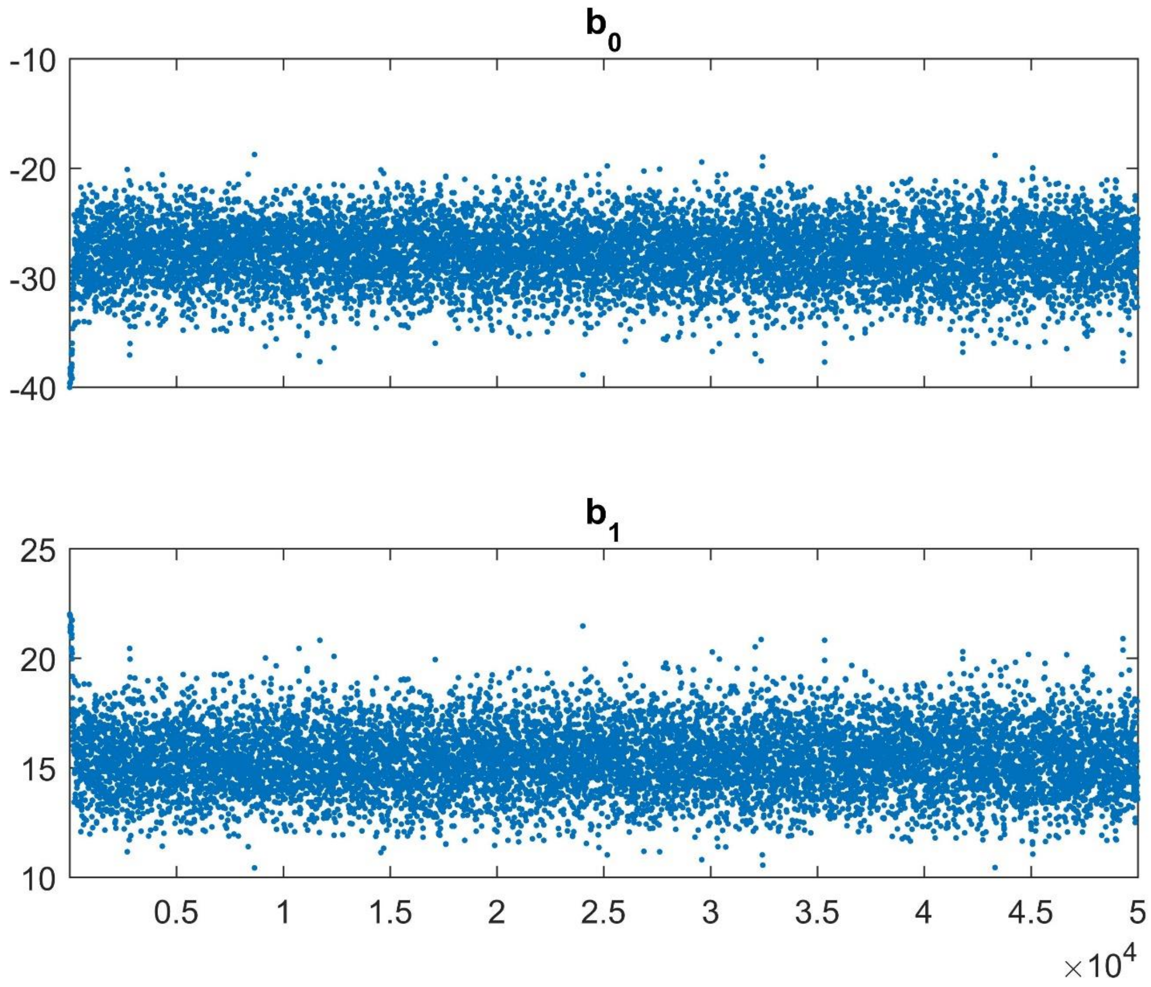


**Supplementary Figure 1.** For Figure 1A, enlarged the "Top right figures: the 1D parameter chains".
